# A Leading Case at Edinburgh

**Published:** 1916-09-30

**Authors:** 


### A LEADING CASE AT EDINBURGH.

The Royal College of Physicians and Surgeons, Edinburgh, brought an action of interdict (injunction) against certain parties using the word " Doctor " or its contraction " Dr.," and pretending that they were doctors or medical practitioners, and using any name or description implying to the public that they were registered under the Medical Acts, and to order the defendants to remove from certain premises in Edinburgh and Glasgow the description of themselves set up there. The first point taken in defence was that the proper procedure was a criminal prosecution under the Medical Acts; and as that was prescribed as the statutory remedy, a further civil remedy such as was now sought was incompetent. The judge of first instance held that injunction was competent even where another statutory remedy had been enacted. In this he followed previous decisions on the question in England and Ireland. But he went on to find that in order to make the civil remedy competent, the plaintiff must aver that he is sustaining actual pecuniary or patrimonial loss. The plaintiffs, however, argued that they had a title to raise the action with the view of protecting the rights of the public, which were being infringed by the defendants. His Lordship, however, could not admit such a claim, pointedly remarking that the Royal College of Physicians was simply a corporation entitled to speak and act for itself and for no other person. Next, the plaintiffs contended that, in fact, they were sustaining loss by the unprofessional actings of the defendants. Their averments in that respect were held relevant, and the case was sent to trial, but only so far as regards Edinburgh, outside of which, it was held, plaintiffs had no actual interest. An appeal was taken to the Second Division of the Court of Session, which adhered to the judgment of the lower Court. Thereupon defendants, recognising apparently that further opposition was futile, withdrew their defences, and consented to perpetual interdict against themselves being given as the judgment of the Court.

